# Ambient Air Pollution and Hospital Admissions of AECOPD in 10 Regions of China: A Self-Controlled Study Based on a Cohort

**DOI:** 10.1021/envhealth.5c00439

**Published:** 2026-01-23

**Authors:** Lu Chen, Yuxuan Zhao, Jun Lv, Dianjianyi Sun, Pei Pei, Ling Yang, Yiping Chen, Huaidong Du, Shichun Yan, Dan Schmidt, Junshi Chen, Zhengming Chen, Liming Li, Canqing Yu

**Affiliations:** † Department of Epidemiology and Biostatistics, School of Public Health, 12465 Peking University, Beijing 100191, China; ‡ 540412 Peking University Center for Public Health and Epidemic Preparedness & Response, Beijing 100191, China; § Key Laboratory of Epidemiology of Major Diseases (Peking University), Ministry of Education, Beijing 100191, China; ∥ State Key Laboratory of Vascular Homeostasis and Remodeling, Peking University, Beijing 100191, China; ⊥ Clinical Trial Service Unit & Epidemiological Studies Unit (CTSU), Nuffield Department of Population Health, 6396 University of Oxford, Oxford OX3 7LF, United Kingdom; # Heilongjiang Center for Disease Control and Prevention, Harbin 150001, China; ∇ 442518 China National Center for Food Safety Risk Assessment, Beijing 100022, China

**Keywords:** air pollution, chronic obstructive pulmonary disease, acute exacerbation, case-crossover design, self-controlled case-series design

## Abstract

Gaps exist concerning the associations of PM_2.5–10_, gaseous pollutants, and composite pollutants with the acute exacerbation of chronic obstructive pulmonary disease (AECOPD). The interaction effects of lifestyle factors, indoor air pollution, and meteorological conditions with air pollutants have been underexplored. This time-stratified case-crossover study was nested within the China Kadoorie Biobank cohort study of middle-aged and elderly participants from five urban and five rural areas in China spanning from 2004 to 2018. 10,712 participants were included, each with a mean of 2.3 episodes of AECOPD hospitalizations during the observation period. A composite air pollution score was derived through principal component analysis. **S**hort-term exposure to particulate matter and NO_2_ was positively associated with hospital admissions for AECOPD, with the largest temperature- and humidity-adjusted odds ratio (95% confidence interval) of 1.101 (1.073, 1.129) at a lag of 1 day for per 1 standard deviation increase in air pollution score. The effect of O_3_ appeared to be conflicting. The associations were further corroborated by the self-controlled case-series design. Unfavorable body shapes, cold season, and low humidity could exacerbate the influence of air pollution. These findings reinforce the evidence of links between air pollution and AECOPD and implicate the management of body weight and cold and dry circumstances.

## Graphical abstract

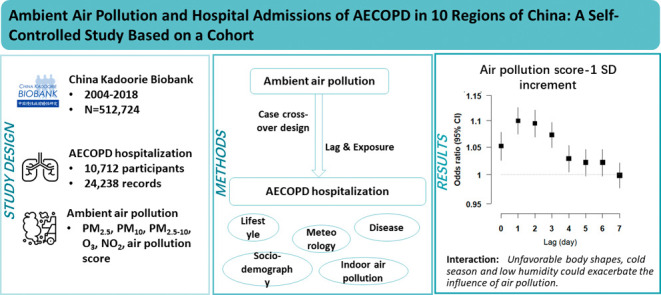

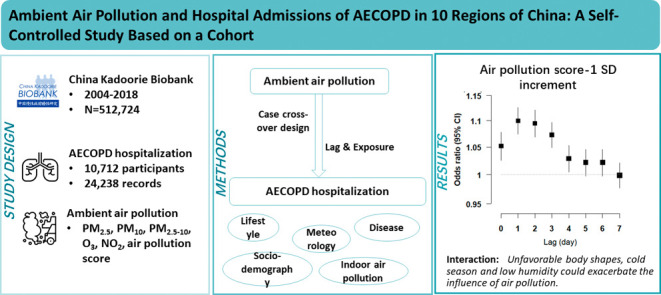

## Introduction

Ambient air pollution, primarily attributable to particulate matter (PM) with an aerodynamic diameter of ≤2.5 μm (PM_2.5_), was estimated to result in 4.2 million premature deaths of cardiovascular and respiratory diseases and cancer worldwide in 2019.^1^ It constitutes a major environmental health concern, especially within the World Health Organization (WHO) South-East Asia and Western Pacific Regions.^1^

Emerging evidence shows that air pollution may be associated not only with noncommunicable diseases but also with acute attacks of diseases, including the acute exacerbation of chronic obstructive pulmonary disease (AECOPD).^2–6^ However, few studies distinguish the effects of PM_2.5–10_ from PM_10_, and the independent role of PM_2.5–10_ remains poorly understood. Also, the findings regarding the associations of gaseous pollutants with AECOPD were inconsistent.^2–5^ This discrepancy may be attributed to limited variations in air pollution concentrations, especially in developed countries or regions, given that many studies were confined to single administrative divisions or specific geographical areas.^3,4^ To enhance our understanding of the impact of air pollution on AECOPD, a study investigating and comparing multiple locations is merited. While the comprehensive assessment of health impacts from multiple air pollutants has garnered increasing recognition in environmental epidemiology,^7,8^ to date, no study has systematically examined the combined short-term effects of multipollutant exposure on AECOPD, to the best of our current scientific understanding.

Most prior studies relied on aggregate data, whereas data at the individual level render an opportunity to examine nuanced effect modifications by personal characteristics. Although some researchers have explored potential effect modifiers (i.e., sociodemographic characteristics and the season of hospital admissions) through stratified analyses, few studies have considered the effect modifications by lifestyle factors and indoor air pollution.^2^ Furthermore, the interaction effects of meteorological conditions with air pollutants have been underexplored, and the existing findings are conflicting.^9^

In the present study, we employed a self-controlled design to examine associations of individual and composite air pollutants with AECOPD across 5 urban and 5 rural regions in China based on the large-scale cohort data from the China Kadoorie Biobank (CKB) study, spanning from 2004 to 2018. We further investigated the interaction effects of multiple pollutants and effect modifiers on AECOPD.

## Methods

### Study Population

The design of CKB has been elaborated upon elsewhere.^10,11^ Briefly, the baseline survey of CKB was initiated during 2004–2008, enrolling ∼0.5 million participants aged 30–79 from 5 urban and 5 rural areas. The China Kadoorie Biobank (CKB) complies with all of the required ethical standards for medical research on human subjects. Ethical approvals were granted and have been maintained by the Chinese Centre for Disease Control and Prevention (Beijing, China: 005/2004) and the University of Oxford (UK: 025–04). Written informed consent was procured from each participant involved in the study after a detailed introduction of the research at baseline.

### Baseline Data Collection

During the baseline survey, about 1800 study clinics were set near participants’ residences to ensure convenience (most within a 1 km radius). Qualified staff conducted interviews with participants to collect information about sociodemographic characteristics, lifestyle factors, indoor air pollution, and personal medical history. Detailed information on smoking included smoking status (never, ever, or currently) and the primary reason for smoking cessation in quitters. The main fuel types for cooking and heating were classified into 2 major categories: solid and nonsolid. The frequency of direct exposure to second-hand smoke (SHS) either at home or in the workplace was inquired.

Physical measurements, including height, weight, waist circumference (WC), forced expiratory volume in 1 s (FEV1), and forced vital capacity (FVC), were performed by trained technicians using calibrated instruments. Body mass index (BMI) was derived as the ratio of weight (kg) to the square of the baseline height (m). FEV1 and FVC were assessed through two successful expiratory maneuvers, and a larger measurement was adopted.

### Exposure and Covariate Assessment

Daily concentrations of PM_2.5_, PM_10_, 8h O_3_, and NO_2_ were retrieved from the ChinaHighAirPollutants data sets.^12^ PM_2.5–10_ level was estimated by subtracting PM_2.5_ from PM_10_. The air pollution score was calculated as the first principal component (PC) by principal components analysis (PCA), and standardized with a mean of 0 and a standard deviation (SD) of 1. Additionally, daily 2-m temperature and specific humidity were extracted from NASA GESDISC DATA.^13^ Participants were assigned ambient exposure assessments based on the study clinics where they were recruited. The details of assessments are described in the Supporting Information.

### Outcome Ascertainment

Incident outcome cases were primarily identified by electronic linkage, via a unique personal identification number, to the inpatient health insurance (HI) database. Hospitalizations that occurred during the follow-up and had a discharge diagnosis of AECOPD (coded as J44.0-J44.1 by the International Classification of Diseases, 10th revision) were extracted from the HI data. By December 31, 2018, 24,409 incident AECOPD cases involving 10,727 participants were documented since the baseline survey.

### Study Design

A time-stratified case-crossover design was employed to estimate the associations of exposure to air pollutants with hospital admissions for AECOPD. The design was initially proposed by Maclure to study the effects of transient exposures on the abrupt onset of events^14^ and then generalized to the context of air pollution and acute events.^15^ The self-matched design compares each case’s exposure levels during hazard periods relevant to the causation of the outcome with one or more control periods.^14,15^ This method eliminates control selection bias and confounding led by time-invariant characteristics, thus enhancing efficiency.^14,15^ The time-stratified approach selects control periods based on the day-of-week (DOW) within the same year and month as case days, namely, the day on which the AECOPD hospital admission occurred, thereby accounting for the long-term and seasonal trend, as well as the influence of DOW, ensuring minimal bias.^16^

### Statistical Analysis

Associations of exposure to individual air pollutants with hospital admissions for AECOPD were estimated using conditional logistic regression models. According to previous studies, models were adjusted for the natural cubic splines of the 3-day average temperature and humidity, from the index date to the two preceding days, with degrees of freedom (df) of 6 and 3, respectively.^2,17^ Considering the self-matched design, adjustments were not made for relatively stable factors in the short term, such as sociodemographic factors, lifestyles, and health status. Instead, the specific modifying effects of these characteristics on associations were thoroughly examined, leveraging the detailed profile of the cohort participants. To uncover potential lag patterns, exposures of single-day lag over 0–7 days were included in the models, and the single-day lag yielding the largest effect estimate was then selected as the main indicator for quartile and subgroup analyses.

Also, we conducted subgroup analyses to explore whether the association of air pollution with AECOPD hospitalization was consistent across various subpopulations. These subpopulations were stratified by sociodemographic characteristics, lifestyle factors, indoor air pollution, disease status, season of hospital admission, and meteorological conditions. The interaction effects were examined using likelihood ratio tests by comparing models with and without interaction terms for air pollutants and strata variables. To maximize the potential for discovering novel relationships, we opted not to apply multiple testing corrections when exploring potential effect modifiers.

To ensure the robustness of our findings, we conducted several sensitivity analyses. First, we adopted the self-controlled case-series (SCCS) design to confirm the relationship between exposure to air pollutants and hospital admissions for AECOPD, further considering the heterogeneity across the 10 cities. The observation period from enrollment to censoring for each case was split day by day. Also, the occurrence of events was regressed on the air pollutants on a continuous scale each day. The advantages of SCCS resemble the case-crossover design. By contrast, SCCS builds on the principles of a cohort study, focusing on intrapersonal comparisons of each case’s outcome risk during exposure periods to the risk during baseline periods, thereby yielding estimates of relative incidence.^18,19^ The SCCS model requires certain assumptions to produce valid and unbiased estimates. One assumption requires that recurrences of AECOPD events are independent;^19^ therefore, only first hospital admissions of AECOPD were included, and participants with self-reported or screened COPD at baseline were excluded. Another criterion to be met is that the event does not affect subsequent exposure,^19^ and thus deaths within 28 days of AECOPD events were further excluded. Here, a two-stage analysis was conducted to examine both city-specific and combined associations.^20^ At the first stage, conditional logistic models with adjustments for age effect, temperature, humidity, month, and DOW were conducted at the individual regional level. At the second stage, meta-analysis was used to combine the effect estimates from the 10 cities to obtain the short-term health effects of air pollution at the pooled level. Second, pollutants were further mutually adjusted to eliminate potential confounding by coexposures, except for the pair of PM_2.5_ and PM_10_, considering the high correlation (Table S1). Third, the models were adjusted for the natural cubic splines of the 7-day average temperature and humidity. Fourth, relative humidity was included in the models instead of specific humidity. Fifth, the air pollution score was calculated using the equation^21^: $\mathrm{air pollution score}=\frac{\sum_{i=1}^{n}\left({\mathrm{PC}}_{i}E_{i}\right)}{\sum_{i=1}^{n}E_{i}}$ , where PC*_i_* was the PC for which the corresponding eigenvalue (*E_i_*) was ≥1. Sixth, recurrent cases of AECOPD within 28 days of the last acute exacerbation were excluded from the analysis. The robustness of the findings was directly tested according to the difference in estimates of ORs and statistical significance for ORs.

We used Stata (ver. 17.0) and the SCCS package in R software (ver. 4.3.2) for statistical analyses. Statistical significance was defined as the two-sided *P*-values <0.05.

## Results

### Basic Characteristics

The primary analysis included 10,712 participants, each with a mean of 2.3 episodes of AECOPD hospitalizations during the observation period (Figure S1). Of these participants, the mean (SD) age was 61.6 (8.7) years, and 49.0% were female. Compared with the whole CKB participants, AECOPD cases included in the study were generally older, predominantly male, resided in rural areas, had lower educational attainment, smoked regularly, and primarily used solid fuels for cooking. Notably, 58.1% of these hospital admissions occurred during the cold season (October–March next year) (Table 1).

**Table 1:** Baseline Characteristics of AECOPD Cases during the Study Period^a^

variables	AECOPD cases (*N* = 10,712)	whole CKB cohort (*N* = 512,724)
age, years	61.6 (8.7)	52.0 (10.7)
female	5245 (49.0%)	302,519 (59.0%)
urban residents	2014 (18.8%)	226,191 (44.1%)
middle school or above	2189 (20.4%)	252,359 (49.2%)
agriculture/factory worker	7236 (67.6%)	286,319 (55.8%)
currently smoking^b^	5263 (49.1%)	150,804 (29.4%)
BMI, kg/m^2^	22.4 (3.7)	23.7 (3.4)
WC, cm	78.0 (10.5)	80.3 (9.8)
solid fuel for cooking	5542 (51.7%)	185,266 (36.1%)
solid fuel for heating	2999 (28.0%)	187,805 (36.6%)
exposure to SHS 6–7 d/w	5110 (47.7%)	212,970 (41.5%)
self-reported physician-diagnosed asthma	335 (3.1%)	2806 (0.5%)
cold (October–March next year) season at hospital admission^c^	14,092 (58.1%)	

a Continuous and categorical characteristics are presented as mean (SD) and count (percentage), respectively. Abbreviations: AECOPD: acute exacerbation of chronic obstructive pulmonary disease; BMI: body mass index; WC: waist circumference; SHS: second-hand smoke; SD: standard deviation.

b Ex-smokers who had stopped smoking for illness were categorized as current smokers.

c All recurrent cases included.

The distributions of air pollutants showed prominent regional disparities and seasonal variations. Haikou recorded the lowest levels of particulate matter and NO_2_; the pollution from particulate matter was the most severe in Henan; and Zhejiang was high in O_3_ and NO_2_. The particulate pollution and NO_2_ levels peaked in the winter and hit bottom in the summer, whereas the opposite trend in fluctuation was found for O_3_ (Figure S2). The concentration of O_3_ was negatively correlated with PM_2.5_, PM_10_, and NO_2_. Generally, low to moderate correlations were observed among these air pollutants, except for the correlation between PM_2.5_ and PM_10_ concentrations (*r* = 0.948) (Table S1).

The difference between the case and control days reached the maximum at lag 1, 1, 1, 2, and 0 days for PM_2.5_, PM_10_, PM_2.5–10_, O_3_, and NO_2_, respectively. The mean (SD) concentrations of air pollutants on the case days with the maximum difference were 58.1 (33.2) μg/m^3^ for PM_2.5_, 92.3 (46.0) μg/m^3^ for PM_10_, 34.1 (18.5) μg/m^3^ for PM_2.5–10_, 81.9 (33.3) μg/m^3^ for O_3_, and 36.0 (15.5) μg/m^3^ for NO_2_. On the control days, the corresponding values were 57.3 (33.1), 90.6 (44.7), 33.3 (16.9), 81.4 (33.3), and 35.7 (15.3) μg/m^3^, respectively. The temperature on the case days approximated to that on the control days. Specific humidity on the case days was slightly lower than that on the control days (Table 2).

**Table 2:** Distribution of Air Pollutants and Meteorological Conditions on the Case and Control Days^a^

indicator	case/control day	lag 0	lag 1	lag 2	lag 3	lag 4	lag 5	lag 6	lag 7
PM_2.5_, μg/m^3^	case day	57.9 (32.5)	58.1 (33.2)	58.2 (33.7)	58.1 (33.2)	58.0 (33.0)	57.9 (33.1)	57.7 (32.9)	57.6 (33.0)
	control day	57.4 (33.1)	57.3 (33.1)	57.4 (33.1)	57.5 (32.8)	57.6 (33.0)	57.7 (33.2)	57.6 (33.0)	57.6 (33.2)
PM_10_, μg/m^3^	case day	91.5 (44.3)	92.3 (46.0)	92.4 (46.8)	92.1 (45.5)	91.6 (44.8)	91.5 (45.1)	91.4 (44.7)	91.2 (44.8)
	control day	90.7 (44.9)	90.6 (44.7)	90.8 (44.7)	90.9 (44.6)	91.1 (44.7)	91.1 (44.9)	91.0 (44.8)	91.1 (45.2)
PM_2.5–10_, μg/m^3^	case day	33.7 (17.0)	34.1 (18.5)	34.2 (19.2)	34.0 (18.1)	33.6 (17.1)	33.6 (17.3)	33.7 (17.3)	33.5 (17.2)
	control day	33.3 (17.2)	33.3 (16.9)	33.4 (16.8)	33.4 (17.0)	33.5 (17.0)	33.5 (17.0)	33.4 (17.1)	33.5 (17.3)
O_3_, μg/m^3^	case day	81.5 (33.5)	81.9 (33.4)	81.9 (33.3)	81.7 (33.3)	81.9 (33.0)	81.6 (33.2)	81.4 (33.1)	81.2 (33.0)
	control day	81.6 (33.2)	81.6 (33.2)	81.4 (33.3)	81.4 (33.3)	81.5 (33.4)	81.5 (33.4)	81.5 (33.2)	81.3 (33.2)
NO_2_, μg/m^3^	case day	36.0 (15.5)	36.1 (15.5)	36.1 (15.6)	36.1 (15.6)	36.0 (15.6)	36.0 (15.6)	35.9 (15.5)	35.9 (15.4)
	control day	35.7 (15.3)	35.8 (15.3)	35.8 (15.4)	35.8 (15.3)	35.9 (15.4)	36.0 (15.4)	36.0 (15.5)	35.9 (15.4)
temperature, °C	case day	13.9 (9.0)	13.9 (9.1)	13.9 (9.1)	13.8 (9.1)	13.8 (9.1)	13.8 (9.1)	13.8 (9.1)	13.8 (9.1)
	control day	13.9 (9.1)	14.0 (9.1)	13.9 (9.1)	14.0 (9.1)	14.0 (9.1)	14.0 (9.1)	14.0 (9.1)	13.9 (9.1)
specific humidity, g/10 kg	case day	92.8 (54.0)	92.6 (54.1)	92.3 (54.2)	92.2 (54.3)	92.2 (54.3)	92.2 (54.2)	92.1 (54.2)	92.2 (54.2)
	control day	93.1 (54.2)	93.1 (54.2)	93.1 (54.2)	93.1 (54.2)	93.1 (54.1)	93.1 (54.1)	93.1 (54.1)	93.1 (54.2)

a Distributions are presented as mean (SD). Abbreviation: PM_2.5_: particulate matter with an aerodynamic diameter <2.5 μm; PM_10_: particulate matter with an aerodynamic diameter <10 μm; PM_2.5–10_: particulate matter with an aerodynamic diameter of 2.5–10 μm; O_3_: ozone; NO_2_: nitrogen dioxide; SD: standard deviation; P_25_: 25th percentile; P_50_: 50th percentile; P_75_: 75th percentile.

### Air Pollution and Hospital Admissions of AECOPD

The second PC was strongly correlated to O_3_, whereas the first PC primarily represented other pollutants (PM_2.5_, PM_2.5–10_, NO_2_) than O_3_. The air pollution score contributed 54.1–54.5% of the total variance over lag 0–7 days (Table S2; other data not shown). As illustrated in Figure 1 and Table S3, the association curves of PM_2.5_, PM_10_, PM_2.5–10_, and NO_2_ and air pollution score with AECOPD hospitalizations displayed an inverted J-shape over a lag period of 0–7 days. The risk of hospital admissions for AECOPD increased at lag 0 days. It persisted for the following 4–5 days, with the largest temperature- and humidity-adjusted odds ratio (95% confidence interval, CI) of 1.016 (1.009, 1.023), 1.016 (1.011, 1.021), 1.043 (1.031, 1.054), 1.054 (1.035, 1.074), and 1.101 (1.073, 1.129) at lag 1, 1, 1, 0, and 1 days, for a 10 μg/m^3^ increase in PM_2.5_, PM_10_, PM_2.5–10_, and NO_2_, and 1 SD increase in air pollution score, respectively. For O_3_, a 10 μg/m^3^ increase initially reduced the risk of hospital admissions at lag 0 days, followed by a rise in risk, with the highest odds ratio (95% CI) of 1.009 (1.002, 1.016) at lag 4 days. Overall, when the air pollutants were divided into quarters, the risk progressively increased across Q2, Q3, and Q4 compared to Q1. All of the estimates for Q4 vs Q1 were statistically significant.

**Figure 1:**
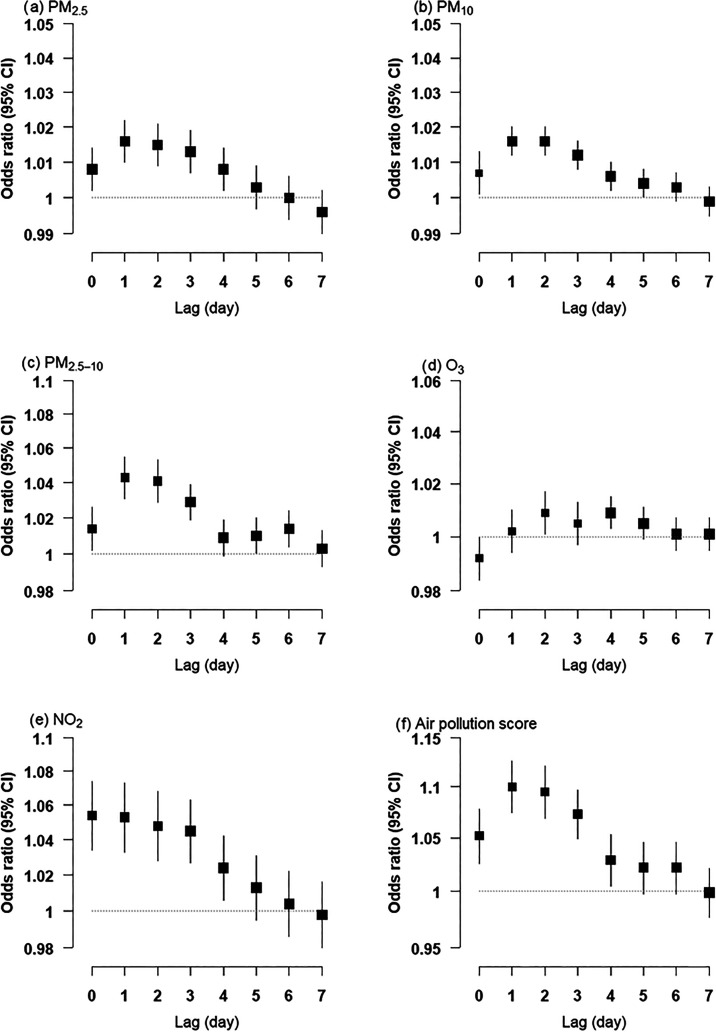
Odds ratios of the adjusted lag-effect between air pollutants and hospital admissions of AECOPD. Abbreviations: AECOPD: acute exacerbation of chronic obstructive pulmonary disease; PM_2.5_: particulate matter with an aerodynamic diameter <2.5 μm; PM_10_: particulate matter with an aerodynamic diameter <10 μm; PM_2.5–10_: particulate matter with an aerodynamic diameter of 2.5–10 μm; O_3_: ozone; NO_2_: nitrogen dioxide; CI: confidence interval. Models were adjusted for the natural cubic splines of the 3-day average temperature and humidity (lag 0–2 days) with 6 and 3 degrees of freedom. The centers of squares represent point estimates for a 10 μg/m^3^ increase in air pollutants or a 1 SD increase in air pollution score, and the lower and upper ends of whiskers represent the lower and upper limits of 95% CI, respectively. The sizes of squares are proportional to precision (1/squares of the standard errors of estimates).

### Subgroup Analyses

The stratified analysis by body shape showed that there was an increased risk of AECOPD hospitalizations associated with PM_2.5_ and PM_10_ among the underweight or obese participants (BMI < 18.5 kg/m^2^, BMI ≥ 28.0 kg/m^2^, or WC ≥ 90 cm for male/≥85 cm for female) compared to those with favorable body shape (*P*_int_ = 0.029 for PM_2.5_ and 0.041 for PM_10_). Further stratification analysis revealed that O_3_ was positively associated with AECOPD hospitalizations only among solid heating fuel users. Also, NO_2_ and air pollution scores were associated with increased risks of AECOPD hospitalizations only during the cold season and under low-humidity conditions. Concerning other characteristics, including sociodemographic characteristics, smoking status, cooking fuel type, SHS exposure, and asthma diagnosis, the associations of air pollution with hospital admissions for AECOPD were consistent across different subpopulations (all *P*_int_ > 0.05) (Table S4).

### Sensitivity Analyses

The associations of air pollution with AECOPD hospitalizations were generally robust to sensitivity analyses (Figure 2 and Table S5). The SCCS design examined the relationship between exposure to air pollutants at lag 0 days and hospital admissions of AECOPD. The findings were consistent with those in the case-crossover design, and estimates were comparable across regions, although estimates were not statistically significant in some regions (heterogeneity *I* ^2^ = 0–48%). Considering the moderate correlation between particulate matter and NO_2_, reductions in the estimates after mutual adjustments were anticipated and deemed acceptable. However, after controlling for other pollutants considered, the effect of PM_2.5_ at lag 1 day and O_3_ at lag 4 day becomes nonsignificant, and the protective effect of O_3_ at lag 0 day becomes more pronounced (not shown; OR = 0.991; 95% CI = 0.983, 0.998). After adjusting for longer durations of temperature and humidity, the risks brought by air pollutants diminished and even to the null for O_3_. Compared with the first PC, the weighted air pollution score took more O_3_ into account and contributed 80.8% of the total variance, and the estimated effect was slightly lower at lag 1 day.

**Figure 2:**
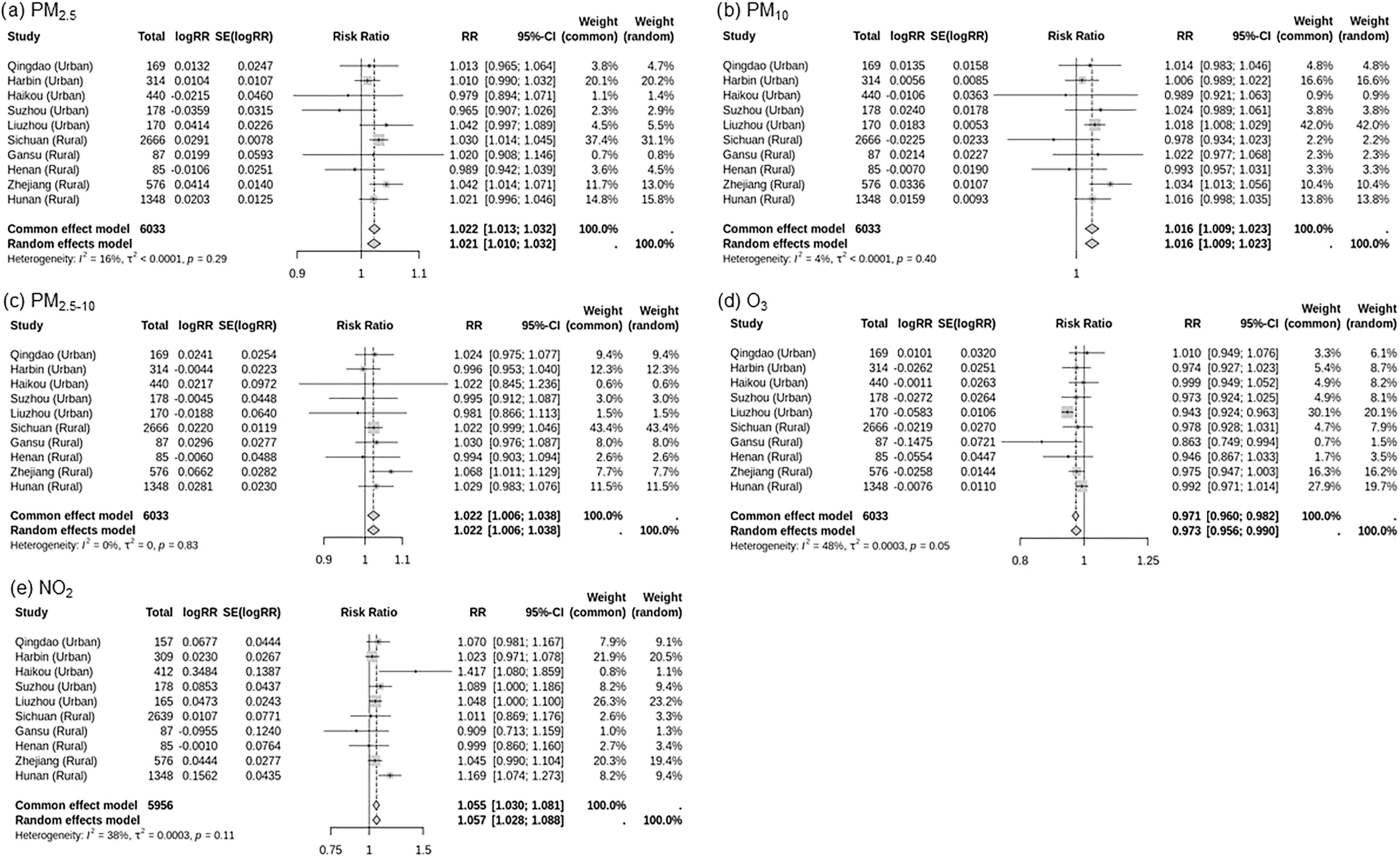
Relative incidence of air pollutants for the first hospital admissions of AECOPD. Abbreviations: AECOPD: acute exacerbation of chronic obstructive pulmonary disease; PM_2.5_: particulate matter with an aerodynamic diameter <2.5 μm; PM_10_: particulate matter with an aerodynamic diameter <10 μm; PM_2.5–10_: particulate matter with an aerodynamic diameter of 2.5–10 μm; O_3_: ozone; NO_2_: nitrogen dioxide; CI: confidence interval; RR: risk ratio. The *I* ^2^ statistic was used to evaluate the heterogeneity of the effect size between cities, with *I* ^2^ > 50% as the existence of heterogeneity. Data on NO_2_ was available since January 1, 2008, and therefore cases occurring prior to January 1, 2008 were excluded.

## Discussion

Based on a large cohort with linkage to inpatient hospitalization records in China, this individual-level case-crossover study demonstrated positive associations of short-term exposure to PM, especially PM_2.5–10_, NO_2_, and air pollution score, with hospital admissions for AECOPD, which could last up to 4–5 days. The findings for O_3_ were conflicting and susceptible to the influence of other factors. Participants with unfavorable body shapes might be more susceptible to PM_2.5_ and PM_10_. Additionally, the cold season and low humidity could potentially exacerbate the influence of air pollution.

The short-term detrimental impacts of PM in triggering acute respiratory events have been well documented in prior studies.^17,22^ One meta-analysis study, which included 14 studies with effect estimates ranging from 0.98 to 1.12, found the risk of AECOPD events, including hospitalization, emergency room visit, and outpatient visit, significantly increased with increments in PM_2.5_ at lag 1 day.^22^ Similar results were observed for PM_10_.^22^ Evidence has shown that PM can induce oxidative stress injury in lung cells.^23^ Both animal models and human cohort studies have linked PM to impaired lung function, accompanied by characteristic inflammatory alterations.^24,25^ The deleterious components of PM, including metals, polyaromatic hydrocarbons, and mineral dust, may contribute to such regulation.^23,26^ Also, our study further strengthened the AECOPD risk posed by PM_2.5–10_, which was underexplored compared to PM_2.5_. Although PM_2.5–10_ generally cannot reach the alveoli, it can enter the bronchi and bronchioles and directly harm the lungs. Future studies can distinguish the effects of different particles on chronic bronchitis-type and emphysema-type COPD.

The effect of NO_2_ and O_3_ on AECOPD has been less thoroughly explored. A few studies indicated that high concentrations of ambient NO_2_ and O_3_ could cause inflammation of the bronchial mucosa, downregulate the bronchoconstriction threshold, and further increase the risk of acute injury to the lung tissue.^27,28^ Nonetheless, the findings remained mixed,^2–5^ and most of the case-crossover studies exhibited no associations of O_3_ with AECOPD.^2,29^ In our study, O_3_ did not have harmful effects until a lag of 2 days. A mortality study based on the Medicare cohort of all beneficiaries aged ≥65 years in the contiguous U.S. from 2000 to 2015 also found that the effects of O_3_ and NO_2_ were reversed in beneficiaries of different characteristics.^20^ Evidence has shown that NO_*x*_ is not only a major precursor of O_3_ in the photochemical reactions but a quencher of O_3_ through NO_*x*_ titration (NO + O_3_ → NO_2_ + O_2_).^30^ Therefore, a negative correlation between NO_2_ and O_3_ was common, especially in heavily polluted areas,^20^ as observed in our study. However, controlling for other pollutants strengthened the protective effect of O_3_ at lag 0 days and diminished the harmful effect at lag 4 days. It is hard to disentangle our findings, and further investigation is required.

Few studies have explored the impact of obesity on the association of air pollution with AECOPD. Our study found that underweight or obese individuals were more susceptible to PM_2.5_ and PM_10_. Low weight was accompanied by a paucity of muscular mass and diminished muscle strength, which were established factors in managing respiratory diseases.^31^ By contrast, excessive fat accumulation, particularly in the abdomen, indicated the oversecretion of bioactive substances, such as inflammatory factors, resulting in structural changes and dysfunction in the body,^32^ as well as limited the capacity and endurance of gas exchange.^33^ A recent study evaluated air pollution-related mortality based on ∼19,000 deceased veterans with prior COPD residing in 25 US metropolitan regions during 2016–2019 and also found that obesity notably increased vulnerability.^34^

The 2025 Global Initiative for Chronic Obstructive Lung Disease (GOLD) report underscored the potential interaction between climate and air quality.^9^ However, the existing evidence is limited and elusive. A study in urban areas of Chengdu, China, from 2015 to 2016, demonstrated stronger associations with COPD hospital admissions at lower temperatures for ambient PM_2.5_ and PM_10_, but not for NO_2_ and O_3_.^35^ A retrospective ecological study, utilizing data on daily emergency hospital admissions to 15 major hospitals in Hong Kong for COPD during 2000–2004, revealed NO_2_ and O_3_ had a greater effect on COPD admissions during the cold season, while no significant difference was identified regarding PM_2.5_ and PM_10_.^36^ The conflicting findings concerning interactions between meteorological conditions and pollutants on AECOPD may partially reflect bias in the personal actual exposure.

To our knowledge, this study constitutes the first epidemiological evidence demonstrating a significant dose–response relationship between a PCA-derived composite air pollution index and AECOPD risk, thereby quantifying synergistic pollutant interactions at environmentally relevant exposure levels. Furthermore, the study entails self-controlled analyses that eliminate confounding and selection bias through time-invariant characteristics. A previous CKB study showed that of the participants who attended both the 2004–2008 baseline survey and the 2013–2014 resurvey, the lifestyles remained largely unchanged,^37^ further supporting the robustness of our case-crossover design spanning only one month. The findings from the two types of self-controlled design adopted further corroborated each other. More than 10 years of follow-up, broad coverage of 10 regions, detailed personal characteristics, and >97% national medical insurance rates also enriched and consolidated the study.

Inevitably, several limitations must be acknowledged. First, participants were assigned exposure levels based on the coordinates of study clinics near their residences, possibly leading to nondifferential misclassification and biasing the association to the null. Exposure during commutes and work was not collected, which may lead to measurement error. However, due to the relatively small variation in pollutant concentrations within regions, this is unlikely to affect our results significantly. Second, indoor ambient exposure may vary between days. In particular, differences in behaviors may affect personal exposure, such as avoiding outdoor activities and using air cleaners on highly polluted days. Nonetheless, this would typically cause an underestimation of the associations. Third, only inpatient admissions for AECOPD events were included in the study. Consequently, mild AECOPD cases without the need for hospitalization admissions were not included, and their association cannot be investigated. Fourth, data on education and occupation were collected only at baseline; thus, we could not assess the impact of their potential changes over time on the observed associations. However, resurveys in a subpopulation have shown that these factors have changed little, and the correlations are moderate to high.

## Conclusion

These findings reinforce the evidence of a link between air pollution and the AECOPD established in regional and local studies, which serves as a solid theoretical basis for implementing efficient interventions and policies for the mitigation of air pollution. This study also implicates the importance of body weight and other enviromental conditions for the management of AECOPD.

## Supplementary Material



## Notes

Supplementary methods; Pearson correlation coefficients among air pollutants and meteorological conditions; eigenvectors and eigenvalues from the principal component analysis eigen decomposition; odds ratios of the adjusted lag-effect between air pollutants and hospital admissions of AECOPD; associations between air pollutants and hospital admissions of AECOPD in the sensitivity analysis; inclusion and exclusion flowchart of participants and records; and daily trends of air pollutants across the 10 regions during 2004–2018 (PDF)
