# Supplementary material for: Ambient Air Pollution and Hospital Admissions of AECOPD in 10 Regions of China: A Self-Controlled Study Based on a Cohort
Source: Environ Health (Wash). 2026 Jan 23;4(6):1176–84. doi: 10.1021/envhealth.5c00439 (PMC13288233; doi:10.1021/envhealth.5c00439)
Supplement: Supplementary file 1 [file eh5c00439_si_001.pdf]

Supporting information

Ambient air pollution and hospital admissions of AECOPD in 10 regions of China: a self-controlled study based on a cohort

Lu Chen[1], Yuxuan Zhao[1], Jun Lv[1,2,3,4], Dianjianyi Sun[1,2,3], Pei Pei[2], Ling Yang[5], Yiping Chen[5], Huaidong Du[5], Shichun Yan[6], Dan Schmidt[5], Junshi Chen[7], Zhengming Chen[5], Liming Li[1,2,3], Canqing Yu[1,2,3¶]

1. Department of Epidemiology and Biostatistics, School of Public Health, Peking University, Beijing 100191, China;
2. Peking University Center for Public Health and Epidemic Preparedness & Response, Beijing 100191, China;
3. Key Laboratory of Epidemiology of Major Diseases (Peking University), Ministry of Education, Beijing 100191, China;
4. State Key Laboratory of Vascular Homeostasis and Remodeling, Peking University, Beijing 100191, China
5. Clinical Trial Service Unit & Epidemiological Studies Unit (CTSU), Nuffield Department of Population Health, University of Oxford, Oxford OX3 7LF, United Kingdom;
6. Heilongjiang Center for Disease Control and Prevention, China;
7. China National Center for Food Safety Risk Assessment, Beijing 100022, China

¶**Corresponding authors:** Canqing Yu, PhD

Email: yucanqing@pku.edu.cn

Catalogue

Supplementary Methods 3

Table S1. Pearson correlation coefficients among air pollutants and meteorological conditions (lag 0 days) 4

Table S2. Eigenvectors and eigenvalues from the principal component analysis eigen decomposition based on the correlation matrix (lag 1 day)..5

Table S3. Odds ratios of the adjusted lag-effect between air pollutants and hospital admissions of AECOPD 6

Table S4. Associations between main lags of air pollutants and hospital admissions of AECOPD by subgroups [a] 7

Table S5. Odds ratios of the adjusted lag-effect between air pollutants and hospital admissions of AECOPD in the sensitivity analysis [*] 12

Figure S1. Inclusion and exclusion flowchart of participants and records in the case-crossover and self-controlled case study..14

Figure S2. Daily trends of air pollutants across the 10 regions during 2004-2018 15

Supplementary Methods

Exposure and covariate assessment

Daily concentrations of $PM_{2.5}$, PM_{10}, and 8h O_3 at a 1×1 km spatial resolution from 2004 to 2018 and NO_2 at a 10×10 km spatial resolution from 2008 to 2018, were retrieved from the ChinaHighAirPollutants datasets,[1] of which technical details have been published elsewhere.[2-5] In summary, artificial intelligence algorithms were trained and applied to estimate daily concentrations based on direct ground monitoring measurements, satellite remote sensing products, and atmospheric reanalysis, demonstrating high accuracy and strong predictive power.[2-5] $PM_{2.5}$ and PM_{10} measurements were derived from the same data source, so $PM_{2.5\text{-}10}$ level was estimated by subtracting $PM_{2.5}$ directly from PM_{10}. And a holistic metric for evaluating combined environmental effects, air pollution score, was calculated as the 1st principal component by principal components analysis (PCA), including $PM_{2.5}$, $PM_{2.5\text{-}10}$, O_3, and NO_2, for each single-day lag over 0~7 days. Additionally, hourly data on 2-meter temperature, specific humidity, and relative humidity during 2004-2018, at a 0.5°×0.625° spatial resolution, were extracted from NASA GESDISC DATA,[6] and 24-h average concentrations were then calculated.

For participants, exposure assessment was conducted at the study clinic level. Initially, the addresses of study clinics were geocoded to obtain longitude and latitude coordinates and then linked to daily data on air pollutants and meteorologic conditions through the coordinates. Consequently, participants were assigned ambient exposure assessments based on the study clinics where they were recruited.

Table S1. Pearson correlation coefficients among air pollutants and meteorological conditions (lag 0 days)

	$PM_{2.5}$	PM_{10}	$PM_{2.5-10}$	O_3	NO_2	Temperature
PM_{10}	0.948					
$PM_{2.5-10}$	0.552	0.789				
O_3	-0.243	-0.145	0.090			
NO_2	0.637	0.657	0.489	-0.200		
Temperature	-0.534	-0.484	-0.238	0.566	-0.532	
Specific humidity	-0.533	-0.531	-0.360	0.423	-0.579	0.927

Abbreviation: $PM_{2.5}$: particulate matter with an aerodynamic diameter < 2.5 μm; PM_{10}: particulate matter with an aerodynamic diameter < 10 μm; $PM_{2.5-10}$: particulate matter with an aerodynamic diameter of 2.5-10 μm; O_3: ozone; NO_2: nitrogen dioxide.

Table S2. Eigenvectors and eigenvalues from the principal component analysis eigen decomposition based on the correlation matrix (lag 1 day)

	$PM_{2.5}$	$PM_{2.5-10}$	O_3	NO_2	Eigenvalue	Cumulative proportion explained, %
PC1	0.60	0.52	-0.19	0.58	2.17	54.1
PC2	-0.06	0.43	0.90	-0.04	1.07	80.8
PC3	-0.04	-0.63	0.33	0.71	0.44	91.7
PC4	0.79	-0.39	0.22	-0.41	0.33	100.0

Abbreviation: $PM_{2.5}$: particulate matter with an aerodynamic diameter < 2.5 μm; PM_{10}: particulate matter with an aerodynamic diameter < 10 μm; $PM_{2.5-10}$: particulate matter with an aerodynamic diameter of 2.5-10 μm; O_3: ozone; NO_2: nitrogen dioxide; PC: principal component. Each row represented the correlation between the original pollutant level and the principal component.

Table S3. Odds ratios of the adjusted lag-effect between air pollutants and hospital admissions of AECOPD

Lag, day	$PM_{2.5}$		PM_{10}		$PM_{2.5-10}$		O_3		NO_2		Air pollution score	
	Odds ratio (95% CI)	*P*	Odds ratio (95% CI)	*P*	Odds ratio (95% CI)	*P*	Odds ratio (95% CI)	*P*	Odds ratio (95% CI)	*P*	Odds ratio (95% CI)	*P*
0	1.008 (1.002, 1.015)	0.015	1.007 (1.002, 1.012)	0.006	1.014 (1.002, 1.025)	0.021	0.992 (0.985, 1.000)	0.040	**1.054 (1.035, 1.074)**	**<0.001**	1.060 (1.033, 1.088)	<0.001
1	**1.016 (1.009, 1.023)**	**<0.001**	**1.016 (1.011, 1.021)**	**<0.001**	**1.043 (1.031, 1.054)**	**<0.001**	1.002 (0.994, 1.010)	0.685	1.053 (1.034, 1.073)	<0.001	**1.101 (1.073, 1.129)**	**<0.001**
2	1.015 (1.009, 1.022)	<0.001	1.016 (1.011, 1.021)	<0.001	1.041 (1.031, 1.052)	<0.001	1.009 (1.001, 1.016)	0.028	1.048 (1.029, 1.067)	<0.001	1.094 (1.068, 1.122)	<0.001
3	1.013 (1.006, 1.019)	<0.001	1.012 (1.008, 1.017)	<0.001	1.029 (1.018, 1.039)	<0.001	1.005 (0.998, 1.012)	0.142	1.045 (1.027, 1.064)	<0.001	1.077 (1.050, 1.103)	<0.001
4	1.008 (1.001, 1.014)	0.026	1.006 (1.001, 1.010)	0.018	1.009 (0.999, 1.020)	0.081	**1.009 (1.002, 1.016)**	**0.008**	1.024 (1.006, 1.042)	0.007	1.032 (1.007, 1.058)	0.012
5	1.003 (0.997, 1.010)	0.330	1.004 (0.999, 1.008)	0.127	1.010 (1.000, 1.020)	0.062	1.005 (0.998, 1.011)	0.151	1.013 (0.996, 1.031)	0.143	1.021 (0.997, 1.047)	0.089
6	1.000 (0.993, 1.006)	0.970	1.003 (0.998, 1.007)	0.241	1.014 (1.004, 1.024)	0.008	1.001 (0.995, 1.008)	0.714	1.004 (0.987, 1.022)	0.616	1.018 (0.993, 1.043)	0.159
7	0.996 (0.990, 1.003)	0.286	0.999 (0.994, 1.003)	0.604	1.003 (0.993, 1.013)	0.595	1.001 (0.994, 1.007)	0.854	0.998 (0.981, 1.016)	0.841	0.995 (0.971, 1.020)	0.701
Main[a]												
Q1	Ref		Ref		Ref		Ref		Ref		Ref	
Q2	1.019 (0.972, 1.069)	0.432	1.005 (0.958, 1.055)	0.838	1.063 (1.016, 1.112)	0.008	1.016 (0.971, 1.063)	0.503	1.027 (0.967, 1.091)	0.387	1.001 (0.947, 1.058)	0.984
Q3	1.058 (1.004, 1.116)	0.037	1.049 (0.994, 1.107)	0.081	1.057 (1.005, 1.111)	0.031	1.091 (1.036, 1.148)	<0.001	1.065 (0.993, 1.142)	0.078	1.042 (0.977, 1.110)	0.213
Q4	1.183 (1.113, 1.258)	<0.001	1.156 (1.087, 1.230)	<0.001	1.132 (1.069, 1.198)	<0.001	1.105 (1.041, 1.173)	0.001	1.150 (1.063, 1.244)	<0.001	1.190 (1.107, 1.280)	<0.001

Abbreviations: AECOPD: acute exacerbation of chronic obstructive pulmonary disease; $PM_{2.5}$: particulate matter with an aerodynamic diameter < 2.5 μm; PM_{10}: particulate matter with an aerodynamic diameter < 10 μm; $PM_{2.5-10}$: particulate matter with an aerodynamic diameter of 2.5-10 μm; O_3: ozone; NO_2: nitrogen dioxide; CI: confidence interval.

Models were adjusted for the natural cubic splines of the 3-day average temperature and humidity (lag 0-2 days) with 6 and 3 degrees of freedom.

[a] The single-day lag yielding the largest estimate was selected as the main indicator for quartile analyses of these four air pollutants, which corresponded to lag 1, 1, 1, 4, 0 and 1 day, respectively.

Table S4. Associations between main lags of air pollutants and hospital admissions of AECOPD by subgroups [a]

Subgroup	Number of cases	$PM_{2.5}$		PM_{10}		$PM_{2.5-10}$		O_3		Number of cases [b]	NO_2		Air pollution score	
		Odds ratio (95% CI)	P_{int}	Odds ratio (95% CI)	P_{int}	Odds ratio (95% CI)	P_{int}	Odds ratio (95% CI)	P_{int}		Odds ratio (95% CI)	P_{int}	Odds ratio (95% CI)	P_{int}
Age at onset, y			0.658		0.626		0.656		0.917			0.100		0.363
<65	6,035	1.013 (1.000, 1.026)		1.015 (1.005, 1.024)		1.042 (1.020, 1.065)		1.006 (0.992, 1.020)		5,939	1.027 (0.987, 1.068)		1.084 (1.031, 1.141)	
≥65	18,203	1.017 (1.009, 1.025)		1.017 (1.011, 1.022)		1.043 (1.030, 1.057)		1.010 (1.002, 1.017)		18,034	1.061 (1.039, 1.084)		1.106 (1.074, 1.139)	
Sex			0.523		0.742		0.731		0.210			0.861		0.821
Male	12,293	1.014 (1.005, 1.023)		1.015 (1.008, 1.022)		1.043 (1.027, 1.060)		1.005 (0.996, 1.014)		12,153	1.054 (1.028, 1.081)		1.097 (1.059, 1.137)	
Female	11,945	1.018 (1.008, 1.028)		1.017 (1.010, 1.024)		1.043 (1.026, 1.059)		1.014 (1.004, 1.024)		11,820	1.055 (1.026, 1.084)		1.106 (1.066, 1.147)	
Urban or rural residents			0.232		0.321		0.730		0.249			0.969		0.328
Rural residents	20,710	1.018 (1.010, 1.026)		1.017 (1.012, 1.023)		1.043 (1.030, 1.056)		1.011 (1.004, 1.018)		20,590	1.054 (1.030, 1.078)		1.107 (1.075, 1.141)	
Urban residents	3,528	1.010 (0.996, 1.023)		1.012 (1.002, 1.023)		1.040 (1.015, 1.065)		1.000 (0.984, 1.016)		3,383	1.053 (1.018, 1.089)		1.080 (1.026, 1.136)	
Educational attainment			0.564		0.340		0.251		0.487			0.731		0.346
Primary school and below	20,129	1.015 (1.007, 1.023)		1.015 (1.010, 1.021)		1.040 (1.027, 1.053)		1.010 (1.003, 1.018)		19,957	1.057 (1.035, 1.080)		1.094 (1.063, 1.127)	
Middle school or above	4,109	1.019 (1.005, 1.033)		1.020 (1.010, 1.030)		1.053 (1.028, 1.078)		1.004 (0.988, 1.020)		4,016	1.044 (1.002, 1.087)		1.120 (1.063, 1.181)	

Subgroup	Number of cases	$PM_{2.5}$		PM_{10}		$PM_{2.5\text{-}10}$		O_3		Number of cases [b]	NO_2		Air pollution score	
		Odds ratio (95% CI)	P_{int}	Odds ratio (95% CI)	P_{int}	Odds ratio (95% CI)	P_{int}	Odds ratio (95% CI)	P_{int}		Odds ratio (95% CI)	P_{int}	Odds ratio (95% CI)	P_{int}
Occupation			0.521		0.589		0.782		0.389			0.850		0.867
Non-agriculture/factory worker	6,686	1.014 (1.002, 1.025)		1.015 (1.006, 1.023)		1.040 (1.020, 1.061)		1.005 (0.993, 1.016)		6,530	1.053 (1.021, 1.085)		1.099 (1.053, 1.148)	
Agriculture/factory worker	17,552	1.017 (1.009, 1.025)		1.017 (1.011, 1.023)		1.043 (1.029, 1.057)		1.011 (1.003, 1.019)		17,443	1.054 (1.029, 1.079)		1.100 (1.066, 1.135)	
Smoking status [c]			0.884		0.630		0.188		0.300			0.486		0.811
Currently smoking	12,514	1.015 (1.005, 1.025)		1.016 (1.009, 1.023)		1.044 (1.028, 1.061)		1.005 (0.996, 1.015)		12,391	1.054 (1.027, 1.082)		1.095 (1.057, 1.135)	
Currently not smoking	11,724	1.017 (1.008, 1.027)		1.017 (1.010, 1.023)		1.041 (1.025, 1.058)		1.013 (1.003, 1.023)		11,582	1.054 (1.026, 1.082)		1.106 (1.067, 1.147)	
BMI, kg/m^2			0.225		0.300		0.658		0.168			0.804		0.424
<18.5 or ≥28.0	5,367	1.020 (1.006, 1.034)		1.018 (1.008, 1.028)		1.042 (1.018, 1.066)		1.014 (1.000, 1.028)		5,303	1.060 (1.021, 1.100)		1.110 (1.054, 1.170)	
18.5-28.0	18,871	1.015 (1.007, 1.022)		1.016 (1.010, 1.021)		1.043 (1.030, 1.056)		1.008 (1.000, 1.015)		18,670	1.052 (1.030, 1.075)		1.097 (1.066, 1.130)	
WC, cm			0.074		0.048		0.158		0.936			0.512		0.049
Male: ≥90/Female: ≥85	4,463	1.026 (1.012, 1.041)		1.024 (1.013, 1.034)		1.052 (1.028, 1.076)		1.011 (0.995, 1.028)		4,391	1.057 (1.015, 1.100)		1.141 (1.080, 1.204)	
Male: <90/Female: <85	19,775	1.013 (1.005, 1.021)		1.014 (1.009, 1.020)		1.040 (1.027, 1.053)		1.008 (1.001, 1.016)		19,582	1.054 (1.032, 1.076)		1.090 (1.059, 1.122)	

Subgroup	Number of cases	$PM_{2.5}$		PM_{10}		$PM_{2.5-10}$		O_3		Number of cases [b]	NO_2		Air pollution score	
		Odds ratio (95% CI)	P_{int}	Odds ratio (95% CI)	P_{int}	Odds ratio (95% CI)	P_{int}	Odds ratio (95% CI)	P_{int}		Odds ratio (95% CI)	P_{int}	Odds ratio (95% CI)	P_{int}
Favorable body shape [d]			0.029		0.041		0.284		0.322			0.694		0.086
No	8,342	1.024 (1.013, 1.035)		1.021 (1.013, 1.029)		1.046 (1.028, 1.065)		1.013 (1.001, 1.024)		8,234	1.056 (1.024, 1.089)		1.123 (1.077, 1.171)	
Yes	15,896	1.011 (1.003, 1.020)		1.013 (1.007, 1.019)		1.040 (1.026, 1.055)		1.007 (0.999, 1.016)		15,739	1.053 (1.028, 1.078)		1.087 (1.053, 1.122)	
Cooking fuel use			0.566		0.632		0.701		0.859			0.148		0.722
Non-solid fuel	10,859	1.015 (1.006, 1.024)		1.016 (1.009, 1.023)		1.042 (1.026, 1.058)		1.010 (1.000, 1.019)		10,679	1.068 (1.041, 1.095)		1.101 (1.063, 1.140)	
Solid fuel	13,379	1.017 (1.007, 1.027)		1.017 (1.010, 1.024)		1.044 (1.027, 1.061)		1.008 (0.999, 1.018)		13,294	1.039 (1.011, 1.068)		1.101 (1.061, 1.143)	
Heating fuel use			0.986		0.481		0.165		<0.001			0.579		0.231
Non-solid fuel	18,385	1.017 (1.009, 1.024)		1.018 (1.013, 1.024)		1.051 (1.038, 1.064)		1.002 (0.994, 1.010)		18,170	1.055 (1.034, 1.077)		1.116 (1.084, 1.148)	
Solid fuel	5,853	1.014 (0.999, 1.028)		1.010 (0.999, 1.020)		1.015 (0.991, 1.040)		1.025 (1.012, 1.038)		5,803	1.057 (1.011, 1.106)		1.051 (0.993, 1.112)	
Exposure to SHS, d/w			0.409		0.199		0.129		0.314			0.692		0.391
≤5	12,364	1.015 (1.006, 1.024)		1.015 (1.008, 1.021)		1.037 (1.022, 1.053)		1.008 (0.999, 1.017)		12,179	1.053 (1.028, 1.079)		1.097 (1.061, 1.135)	
6-7	11,874	1.017 (1.007, 1.028)		1.018 (1.011, 1.026)		1.049 (1.032, 1.067)		1.010 (1.001, 1.020)		11,794	1.056 (1.024, 1.087)		1.106 (1.063, 1.150)	
Asthma diagnosis at baseline			0.837		0.650		0.087		0.209			0.790		0.596

Subgroup	Number of cases	$PM_{2.5}$		PM_{10}		$PM_{2.5\text{-}10}$		O_3		Number of cases [b]	NO_2		Air pollution score	
		Odds ratio (95% CI)	P_{int}	Odds ratio (95% CI)	P_{int}	Odds ratio (95% CI)	P_{int}	Odds ratio (95% CI)	P_{int}		Odds ratio (95% CI)	P_{int}	Odds ratio (95% CI)	P_{int}
No	23,432	1.016 (1.009, 1.023)		1.016 (1.011, 1.021)		1.041 (1.029, 1.053)		1.009 (1.002, 1.016)		23,201	1.054 (1.034, 1.075)		1.099 (1.071, 1.128)	
Yes	806	1.006 (0.977, 1.036)		1.016 (0.994, 1.038)		1.089 (1.027, 1.154)		1.021 (0.986, 1.057)		772	1.042 (0.957, 1.134)		1.114 (0.991, 1.252)	
Season			0.135		0.121		0.041		0.721			0.002		0.044
Warm (April-September)	10,146	1.004 (0.986, 1.023)		1.009 (0.998, 1.021)		1.026 (1.005, 1.048)		1.008 (0.999, 1.017)		10,018	0.994 (0.953, 1.037)		1.043 (0.982, 1.108)	
Cold (October-March next year)	14,092	1.017 (1.010, 1.025)		1.018 (1.012, 1.023)		1.049 (1.036, 1.063)		1.008 (0.998, 1.019)		13,955	1.070 (1.048, 1.092)		1.114 (1.083, 1.145)	
Temperature			0.942		0.425		0.064		0.611			0.002		0.103
Low	-	1.017 (1.009, 1.024)		1.018 (1.013, 1.024)		1.053 (1.040, 1.066)		1.015 (1.002, 1.028)		-	1.069 (1.047, 1.092)		1.120 (1.089, 1.152)	
High	-	1.019 (1.003, 1.035)		1.014 (1.004, 1.024)		1.028 (1.008, 1.048)		1.012 (1.004, 1.020)		-	1.021 (0.981, 1.062)		1.072 (1.015, 1.133)	
Specific humidity			0.586		0.159		0.070		0.931			0.034		0.068
Low	-	1.015 (1.008, 1.023)		1.018 (1.012, 1.023)		1.051 (1.038, 1.063)		1.011 (0.998, 1.024)		-	1.064 (1.042, 1.086)		1.113 (1.082, 1.145)	
High	-	1.013 (0.997, 1.030)		1.011 (1.000, 1.022)		1.024 (1.002, 1.047)		1.008 (1.000, 1.016)		-	1.027 (0.987, 1.068)		1.053 (0.994, 1.116)	

Abbreviations: AECOPD: acute exacerbation of chronic obstructive pulmonary disease; $PM_{2.5}$: particulate matter with an aerodynamic diameter < 2.5 μm; PM_{10}: particulate matter with an aerodynamic diameter < 10 μm; $PM_{2.5\text{-}10}$: particulate matter with an aerodynamic diameter of 2.5-10 μm; O_3: ozone; NO_2: nitrogen dioxide; CI: confidence interval; BMI: body mass index; WC: waist circumstance; SHS: second-hand smoke.

Models were adjusted for the natural cubic splines of the 3-day average temperature and humidity (lag 0-2 days) with 6 and 3 degrees of freedom, where appropriate.

[a] The single-day lag yielding the largest estimate was selected as the main indicator for quartile analyses of these air pollutants, which corresponded to lag 1, 1, 1, 4, 0, and 1 day, respectively.

[b] Data on NO_2 was available since Jan 1st 2008, and therefore cases occurring prior to Jan 1st 2008 were excluded.

[c] Ex-smokers who had stopped smoking for illness were categorized into current smokers.

[d] BMI: 18.5-28.0 kg/m^2 & WC: <90 cm for male or <85 cm for female.

[*] Divided by medians: low: < P_{50}, high: ≥P_{50}; the number of cases varied because of all negative or only positive outcomes in certain subgroups. Number of cases: Low temperature: PM: 9,610; O_3: 9,743; NO_2: 9,489; air pollution score: 9,539; High temperature: PM: 9,335; O_3: 9,221; NO_2: 9,326; air pollution score: 9,194; Low humidity: PM: 9,010; O_3: 9,434; NO_2: 8820; air pollution score: 8,931; High humidity: PM: 8,723; O_3: 8,627; NO_2: 8,733; air pollution score: 8,589.

Table S5. Odds ratios of the adjusted lag-effect between air pollutants and hospital admissions of AECOPD in the sensitivity analysis *

Exposure	Sensitivity analysis	Odds ratio (95% CI)	*P*
$PM_{2.5}$	Basic model	1.016 (1.009, 1.023)	<0.001
	Additionally adjust for $PM_{2.5\text{-}10}$	1.005 (0.998, 1.013)	0.175
	Additionally adjust for O_3	1.016 (1.009, 1.023)	<0.001
	Additionally adjust for NO_2	1.008 (1.000, 1.016)	0.038
	Additionally adjust for $PM_{2.5\text{-}10}$, O_3, and NO_2	0.999 (0.990, 1.007)	0.999
	Adjust for the 7-day average temperature and humidity	1.012 (1.006, 1.019)	<0.001
	Adjust for relative humidity rather than specific humidity	1.016 (1.009, 1.023)	<0.001
	Recurrent hospitalizations dated within 28 days of last hospitalizations excluded	1.013 (1.006, 1.020)	<0.001
PM_{10}	Basic model	1.016 (1.011, 1.021)	<0.001
	Additionally adjust for O_3	1.016 (1.011, 1.021)	<0.001
	Additionally adjust for NO_2	1.012 (1.007, 1.018)	<0.001
	Additionally adjust for O_3 and NO_2	1.012 (1.007, 1.018)	<0.001
	Adjust for the 7-day average temperature and humidity	1.013 (1.009, 1.018)	<0.001
	Adjust for relative humidity rather than specific humidity	1.016 (1.011, 1.021)	<0.001
	Recurrent hospitalizations dated within 28 days of last hospitalizations excluded	1.012 (1.007, 1.018)	<0.001
$PM_{2.5\text{-}10}$	Basic model	1.043 (1.031, 1.054)	<0.001
	Additionally adjust for $PM_{2.5}$	1.038 (1.026, 1.052)	<0.001
	Additionally adjust for O_3	1.043 (1.031, 1.055)	<0.001
	Additionally adjust for NO_2	1.036 (1.024, 1.048)	<0.001
	Additionally adjust for $PM_{2.5}$, O_3, and NO_2	1.037 (1.024, 1.050)	<0.001
	Adjust for the 7-day average temperature and humidity	1.036 (1.025, 1.047)	<0.001
	Adjust for relative humidity rather than specific humidity	1.043 (1.031, 1.054)	<0.001
	Recurrent hospitalizations dated within 28 days of last hospitalizations excluded	1.032 (1.020, 1.044)	<0.001
O_3	Basic model	1.009 (1.002, 1.016)	0.008
	Additionally adjust for $PM_{2.5}$	1.008 (1.001, 1.015)	0.021
	Additionally adjust for PM_{10}	1.007 (1.001, 1.014)	0.035
	Additionally adjust for $PM_{2.5\text{-}10}$	1.008 (1.001, 1.015)	0.027
	Additionally adjust for NO_2	1.008 (1.001, 1.015)	0.028
	Additionally adjust for $PM_{2.5}$, PM_{10}, and	1.007 (1.000, 1.015)	0.042

Exposure	Sensitivity analysis	Odds ratio (95% CI)	*P*
	NO_2		
	Adjust for the 7-day average temperature and humidity	1.006 (0.999, 1.013)	0.118
	Adjust for relative humidity rather than specific humidity	1.009 (1.002, 1.015)	0.012
	Recurrent hospitalizations dated within 28 days of last hospitalizations excluded	1.009 (1.002, 1.016)	0.012
NO_2	Basic model	1.054 (1.035, 1.074)	<0.001
	Additionally adjust for $PM_{2.5}$	1.056 (1.034, 1.078)	<0.001
	Additionally adjust for PM_{10}	1.053 (1.031, 1.075)	<0.001
	Additionally adjust for $PM_{2.5\text{-}10}$	1.052 (1.032, 1.072)	<0.001
	Additionally adjust for O_3	1.056 (1.036, 1.075)	<0.001
	Additionally adjust for $PM_{2.5}$, PM_{10}, and O_3	1.055 (1.033, 1.078)	<0.001
	Adjust for the 7-day average temperature and humidity	1.044 (1.025, 1.064)	<0.001
	Adjust for relative humidity rather than specific humidity	1.054 (1.034, 1.073)	<0.001
	Recurrent hospitalizations dated within 28 days of last hospitalizations excluded	1.056 (1.036, 1.077)	<0.001
Air pollution score	Basic model	1.101 (1.073, 1.129)	<0.001
	Adjust for the 7-day average temperature and humidity	1.084 (1.057, 1.112)	<0.001
	Adjust for relative humidity rather than specific humidity	1.101 (1.073, 1.129)	<0.001
	Weighted principal component	1.092 (1.067, 1.117)	<0.001
	Recurrent hospitalizations dated within 28 days of last hospitalizations excluded	1.080 (1.052, 1.109)	<0.001

Abbreviations: AECOPD: acute exacerbation of chronic obstructive pulmonary disease; $PM_{2.5}$: particulate matter with an aerodynamic diameter < 2.5 μm; PM_{10}: particulate matter with an aerodynamic diameter < 10 μm; $PM_{2.5\text{-}10}$: particulate matter with an aerodynamic diameter of 2.5-10 μm; O_3: ozone; NO_2: nitrogen dioxide; CI: confidence interval.

Models were adjusted for the natural cubic splines of the 3-day average temperature and humidity (lag 0-2 days) with 6 and 3 degrees of freedom.

* The single-day lag yielding the largest estimate was selected as the main indicator for sensitivity analyses of these four air pollutants, which corresponded to lag 1, 1, 1, 4, 0, and 1 day, respectively.

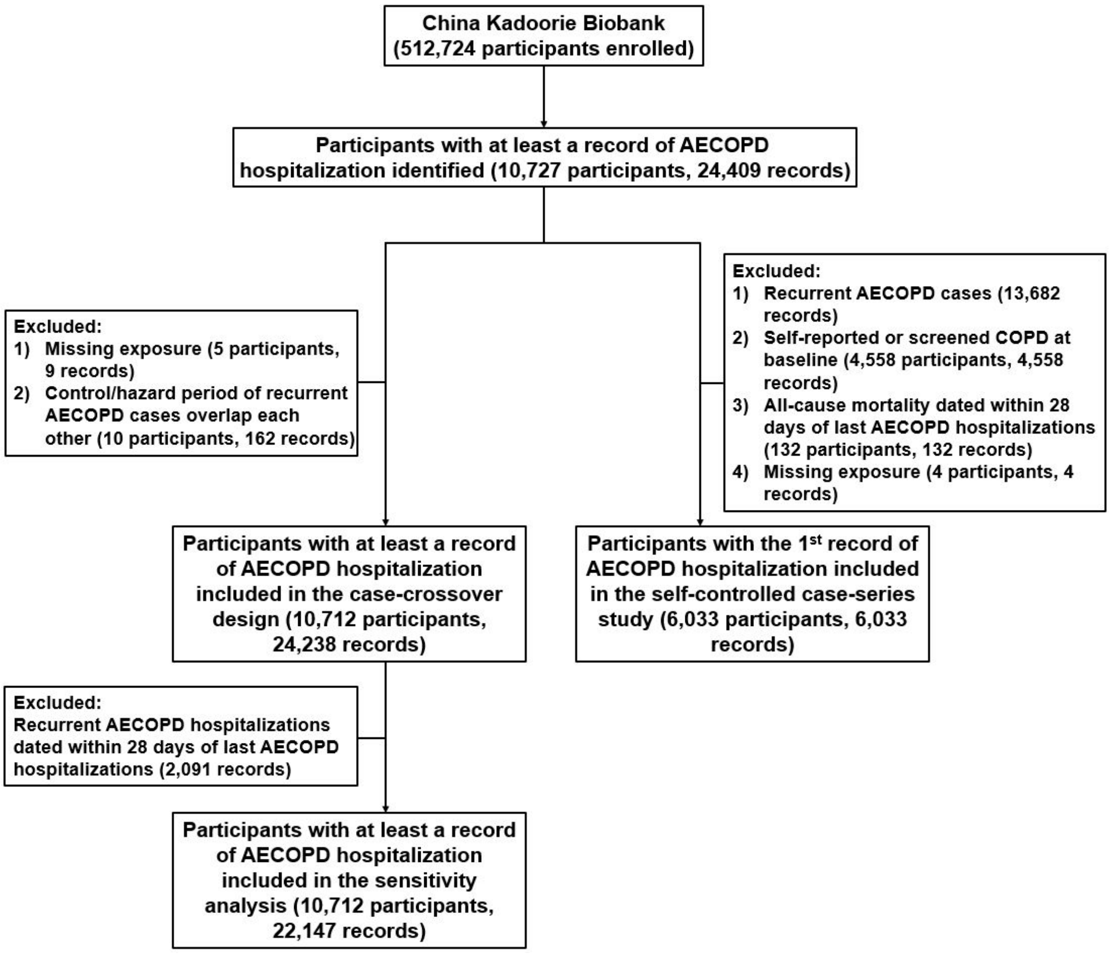

Figure S1. Inclusion and exclusion flowchart of participants and records in the case-crossover and self-controlled case study

Abbreviations: COPD: chronic obstructive pulmonary disease; AECOPD: acute exacerbation of COPD.

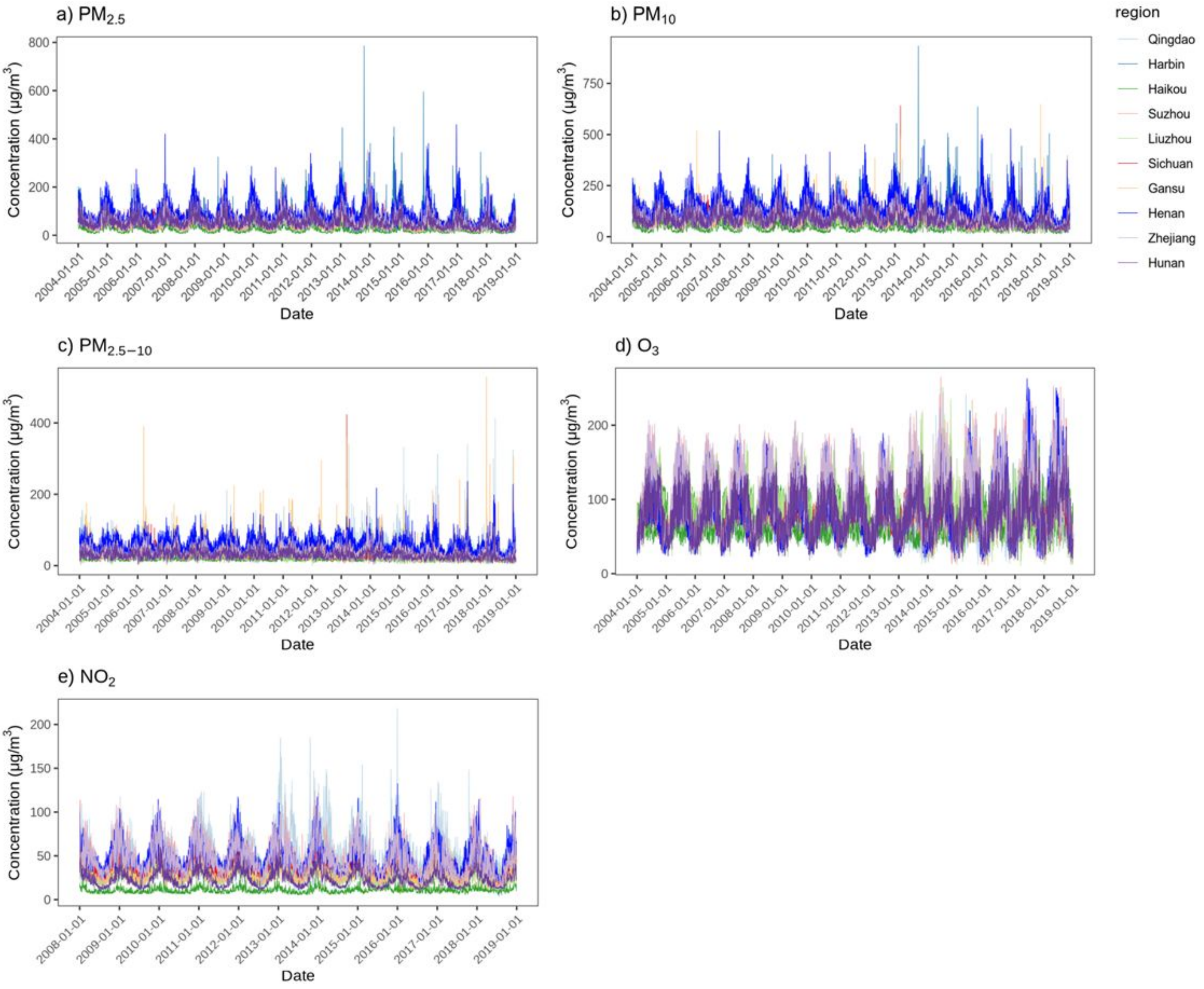

Figure S2. Daily trends of air pollutants across the 10 regions during 2004-2018

Abbreviations: $PM_{2.5}$: particulate matter with an aerodynamic diameter < 2.5 μm; PM_{10}: particulate matter with an aerodynamic diameter < 10 μm; $PM_{2.5-10}$: particulate matter with an aerodynamic diameter of 2.5-10 μm; O_3: ozone; NO_2: nitrogen dioxide.

Data on NO_2 was available since Jan 1st 2008.

Reference

1. Wei J. High-resolution and High-quality Ambient Air Pollutants Dataset for China, 2024.
2. Wei J, Li Z, Lyapustin A, et al. Reconstructing 1-km-resolution high-quality PM2.5 data records from 2000 to 2018 in China: spatiotemporal variations and policy implications. *Remote Sens Environ* 2021;252:112136. doi: https://doi.org/10.1016/j.rse.2020.112136
3. Wei J, Li Z, Xue W, et al. The ChinaHighPM10 dataset: generation, validation, and spatiotemporal variations from 2015 to 2019 across China. *Environ Int* 2021;146:106290. doi: https://doi.org/10.1016/j.envint.2020.106290
4. Wei J, Li Z, Li K, et al. Full-coverage mapping and spatiotemporal variations of ground-level ozone (O3) pollution from 2013 to 2020 across China. *Remote Sens Environ* 2022;270:112775. doi: https://doi.org/10.1016/j.rse.2021.112775
5. Wei J, Li Z, Wang J, et al. Ground-level gaseous pollutants (NO2, SO2, and CO) in China: daily seamless mapping and spatiotemporal variations. *Atmos Chem Phys* 2023;23(2):1511-32. doi: 10.5194/acp-23-1511-2023
6. Gelaro R, McCarty W, Suárez MJ, et al. The Modern-Era Retrospective Analysis for Research and Applications, Version 2 (MERRA-2). In: NASA/GSFC, ed. Greenbelt, MD, USA: NASA Goddard Earth Sciences Data and Information Services Center (GES DISC), 2017.
